# Twenty Sixth Annual Session of the Alabama Dental Association, 1895

**Published:** 1895-06

**Authors:** 


### Twenty Sixth Annual Session of the Alabama Dental Association, 1895.

The Alabama Dental Association convened at the Battle House in its twenty-sixth annual session. The following officers were present: Dr. O. H. Boyd, president, Troy; Dr. O. C. Farrish, first vice-president, Camden; Dr. H. B. Williamson, second vice-president, Evergreen; Dr. G. M. Rosseau, treasurer, Montgomery; Dr. S. W. Foster, secretary, Decatur (now residing in Atlanta, Ga.) There were present about forty members when the session opened in the ladies' ordinary at 11 o'clock.

The meeting was called to order by President Boyd, who introduced Rev. J. J. Taylor, of the Baptist Church of this city, who opened the proceedings with prayer.

The President then introduced Hon. E. M. Robinson, who delivered the address of welcome. Mr. Robinson is a bright young lawyer who enjoyed the distinction of being the youngest member of the late Alabama Legislature, but who is highly regarded, and enjoys quite a reputation as an orator.

Of the advantages of associated effort he said: "Your present annual meeting marks the twenty-sixth anniversary of the Alabama State Dental Association, and I feel safe in the assertion that during the period of its existence, it has done more to dignify and elevate your profession than any other power or influence. The value and potency of organized endeavor cannot be overestimated in this busy, bustling universe of ours, and right wisely have the dentists of Alabama recognized the force of this great principle. No science, no profession, no calling or occupation that ever invoked the energy or ingenuity of man, has ever yet reached the highest point of possible achievement. However, much has been accomplished in the past, and to whatever extent the torch of genius has lighted the pathway of progress, there are always new regions to explore, new discoveries to add to the splendor of past accomplishment. And the science in which your efforts are enlisted is no exception to this universal rule. . .

The most effective method for raising the standard of any profession is the comparison of ideas, the interchange of useful thoughts. That is the function of your annual convention, and its influence is felt throughout the dental world. You have met, not to elect your officers for the ensuing year and then adjourn, but for the purpose of promoting the cause of dental science. The lessons which you have learned in the school of experience, beside the dental chair, will be imparted to your brother dentists in your deliberations here. The thoughts moulded by each of you in the silent solitude of your studios will now be published to the world and will doubtless enrich the dental literature of your age."

Of the early history of dentistry, and its recent advances he said: "Dentistry does not look to the nineteenth century for its origin and birth. Way back in the early morning of civilization, when the star of empire was still hovering in oriental skies, and when the land of the pyramids was the first in historic importance, the treatment of the teeth was a recognized branch of Egyptian science; and more than a century ago, the supplanting of nature's handiwork with artificial masticators was granted place in your professional equipment. But, like every other science or profession whose votaries are worthy of the name, dentistry has kept pace with the progress of the ages, has stood abreast of civilization's ceaseless march, and to-day registers the highest point in its development.

Fifty years ago when a dentist in the hills of the "Nutmeg state" startled and electrified the dental and medical world by the discovery of anæsthesia, and its application to the practice of dentistry, I have no doubt there were those who believed that your profession had arrived at the flood tide of its glory, and that no further advancement was possible. And yet, during the last half century, the dentists of America have done more to perfect the science of dentistry than had been achieved in all its previous history."

He paid an eloquent tribute to the services rendered to humanity by the science of dentistry, as follows: "The dentist of to-day is essentially a many-sided man. As the stately river draws its coursing current from a thousand hills and dales, and is fed by myriad rippling rivulets, so does the science of dentistry receive its contributions from well nigh every source of human thought.

Yours is indeed a lofty profession. You alleviate human ills, banish the most excruciating of earthly pains, and, with the magic wand of science, you level the mountains of physical affliction. You apply to the distorted nerves of the suffering patient that subtle substance which destroys the power to feel, you lift him on the wings of morning to ethereal realms, and, while

> "His soul to fancy's fond suggestion yields,
> And roams romantic in her airy fields."

you perform the operation which would formerly have caused him to howl with agony, and then you bid your grateful subject grieve no more.

He who lessens human woe and stimulates human happiness, is termed a benefactor of mankind. And surely you who master the mysteries of the mouth, explore its hidden recesses, and place content and peace to rule where anguish and distress were wont to reign supreme, should wear the benefactor's jewelled crown."

He closed his address with the following glowing, poetic words of welcome: Welcome, then, exponents of a noble science, welcome to Mobile! The "Queen city of the gulf" salutes you and bids you hail! Her streets are yours. The doors of her hospitable homes will open at your bidding. The trees, which fringe her avenues, have blossomed into vernal beauty at your coming, and the fragrant flowers, in recognition of your presence, send forth their sweetest exhalations. The restless waters of her beautiful bay murmur a joyous greeting as they lap the shifting sands away; and the breezes, which bring their messages from o'er the gulf's broad bosom, breathe forth a melody of welcome.

May your deliberations be pleasant and profitable, unmarred by a single detracting incident; may your sojourn among us bring you closer to our people, and establish ties of friendship that shall endure forever; and, in after years, "When memory's bells chime soft and low," may the recollection of your visit to

Mobile steal upon your senses, like a blissful benediction from an unforgotten past.

Welcome, thrice welcome, to Mobile!

In his address in reform to this genial greeting, Dr. S. W. Foster said: "Our hearts were made glad as we listened to these words of greeting so characteristic of Southern hospitality. And we feel as we enter into the deliberations of this meeting, inspired by them to greater enthusiasm, and gentlemen, if we assume this as the pervading spirit during this, the twenty-sixth annual meeting of the Alabama Dental Association, it will be replete with success and we will all return to our homes wiser men and better prepared to supplant the thistle of pain with the poppy of ease."

Sketching briefly the early history of Alabama, he said: "Over her waved the scepter of four different nationalities. Here at the entrance to this beautiful bay, near two hundred years ago, a French colony settled. After sixty years of adversity as well as prosperity it was ceded to Great Britain. And a little more than one year later the Spanish government obtained control and it was, as late as the year 1803 before the United States came into possession of it, and well might she feel proud of such a possession.

Of the early history of the Alabama Dental Association Dr. Foster said: "Just twenty-six years ago a small band, composed of eight members of our profession, men who loved their noble work, men who realized in their hearts what a boon to suffering humanity the science of dentistry was, men whose hearts were ready and hands willing to spare no time or expense, that they might, by their united efforts, grapple scientifically with the diseases of the oral cavity. Those diseases which so effectually destroy the life-giving function of digestion, and bring him, who otherwise would be a stalwart man, to a premature death. These were the men, who saw that there was a higher, a nobler calling than the mere eking out of an existence, or filling their coffers with gold. Our constitution tells us that their design was to promote and foster the advancement of knowledge in dentistry, in course and good will. It was this beneficent and ennobling spirit which called them together in Montgomery for the purpose of organizing the Alabama Dental Association, which to-day stands as a monument to their efforts, and an incentive for us, to push onward to greater achievements. Some of that noble band have since fallen at their post of duty, others have done a noble work and growing weary have resigned their places to younger men, and with their blessings bid them God speed. But I am happy, as I stand before you to-day, to know that he who presides over this meeting was one of those few self-sacrificing men who organized the association and formulated its constitution. I congratulate him this morning, as he occupies the most honored chair within the gift of this society upon the success of the work he aided in putting forth, and to which he has been faithful. The Alabama Dental Association has been a grand success. It has done more to lift our profession from the valley of mediocrity and place it on the high plane of perfection of to-day, than any other similar organization. Through its influence valuable dental laws have been secured and the state dental examining board created. Our dental law and examining board are cited as authority. It has had due influence toward raising the curriculum of our dental colleges. And its blessings bestowed upon humanity are untold. And now, gentlemen, as we again assemble in this capacity, let us with appreciation due him, who has labored for more than a quarter of a century for the good of our association, do honor to the grand old profession, and make this the most instructive meeting during its history."

President Boyd in his annual address said: "It is not my intention, gentlemen, to inflict upon you a lengthy array of pretty words, nor to call your attention to everything that might more properly be discussed by you, for I think the shortest compliance with the constitutional requirement will serve you best.

"Ours, gentlemen, is a grand calling—a calling of labor and forebearance, of vexations and heroisms, that the masses know not of. Grand, because of the masterly effort made to relieve human suffering and make human health and comfort, and grand, because of the scientific research and because of the mechanical and surgical skill displayed. Who is it that can contrast the public estimate of dentistry of a few years ago with that of to-day and not be impressed with the wonderful strides of advancement. It is true that the progress of civilization in general has been greater for the past few years than was ever before known, but with all that none of the researches into the misty mazes of science have been so far reaching, nor so beneficial and comforting as have those made by the dental profession. This progress is due, in great part, to the young men. The same man who said that 'the pen is mightier than the sword' has remarked that in science the young men are the practical elders, inasmuch as they are schooled in the latest experiences gathered up, while their seniors are cramped by the dogmas they were schooled in while the world was some decades younger.

"Had we here in this association older men who are content to live by the theories they gathered up while younger, this statement would be true to the letter. But as it is, with old men who are not old, and who keep up their indefatigable researches with the ardor of young men, and young men who never tire, but are continually trying to give some benefit to the profession and the world, there seems to be a continual struggle for supremacy between the two, with its ultimate object the best good to humanity. I say, gentlemen, let the struggle continue."

Roll call and payment of dues concluded the morning session.

### AFTERNOON SESSION.

The association was called to order at 3:30 P. M. by President Boyd, and a paper was read by Dr. A. A. Pearson, of Montgomery, on "The Treatment of Pulpless Teeth," the discussion of which occupied the entire afternoon.

The discussion was participated in by Drs. J. P. Gray, D. R. Stubblefield, of Nashville, and W. S. Hightower, of Moss Point, who had previously been elected honorary members of the association; also by Drs. T. M. Allen, of Birmingham, F. H. McAnally, of Jasper, O. C. Farrish, of Camden, W. G. Browne, William Crenshaw and C. V. Rosser, of Atlanta, and R. C. Young, of Anniston.

Dr. Pearson's paper was very brief. He said that he had recently been so fortunate as to cure, in a week, a chronic abscess that had been under another man's treatment for a year without benefit.

His treatment was very simple, requiring only a Donaldson broach, a Gates-Glidden drill in the right-angle hand piece, warm carbolized water and a little carbolic acid on the end of a piece of cotton. With this, and nothing more, he cured the abscess and the patient was satisfied. The tooth was a left, lower molar, the anterior root having two canals. Having treated as above, and cleaned out the root canals filled the root canals with a thin paste of oxide of zinc and oil of cloves, and gutta-percha points. In conclusion he said that notwithstanding the claims of many eminent dentists that caries is being prevented or ought to be prevented, he finds as much decay this year as ever before, and decay continues to recur around fillings all the same.

Professor Gray (of the University of Tenn., Dental Department), said that the great trouble with pulpless teeth and abscesses was that they were treated to death. All that is wanted is to get them clean, and let nature do the rest. The hundred and one remedies that are being continually pumped into and through, only serve to aggravate the conditions and continue the irritation. Prof. Gray advocates the iodoform vapor in small root canals.

Dr. T. M. Allen does not think that iodoform has any place in a dental office, because of its intolerable odor, but uses aristol, which vaporizes and recrystallizes in the root canal in the same way that iodoform is used by Prof. Gray, and has no toxic effects.

Dr. F. H. McAnally cited root canals filled with plaster of Paris, nine years ago, which have never given any trouble.

Dr. D. R. Stubblefield (Vanderbilt University), said he would add one essential omitted by Dr. Pearson in the equipment for treating pulpless teeth, and that is brains—brains as well as common sense, in order to adjust the means to the end in view. For the final removal of septic matter from root canals he advocates peroxide of hydrogen.

As an acid is necessary to retain the extra atom of oxygen in combination which also makes it a germicide, the hydrochloric or sulfuric acid alone gives equally good results. He described the different conditions in which pulpless teeth are found, and the necessity for judgment in opening them up. He believes in removing all infected dentine from the root canals, leaving only healthy tooth structure. He uses amalgam in filling root canals, carrying it on a broken off Donaldson drill to the apex, leaving a little button there, no matter what the rest of the canal may be filled with, a very small portion being sufficient to close the apex solidly, using it "as a lock to the back door." He advocates corrosive sublimate for cleaning out root canals. He said: "it is easily used, you can get it anywhere, it is convenient and trustworthy."

One gentleman (whose name your reporter could not catch), treats with complex phenique and fills with paraffine wax. Keeps accurate records, and does not have more than from five to eight abscesses for every one hundred root canals filled since adopting this method.

Dr. W. G. Browne (Atlanta, Ga.) burns out the canals, considering that what fire will not cleanse nothing will, heating any kind of a nerve canal instrument and introducing it very hot. For inaccessible canals file down and bend a fine copper wire, insert and heat with the electric cautery. Expects to find salol very valuable in root filling material.

Dr. Wm. Crenshaw (Atlanta Dental College), wants a root canal filling that can be removed if necessary, and so does not use amalgam. He carries a little oxy-chloride of zinc into the canal and unites a point of gold wire or gutta percha sprig. Believes that oxy-chloride of zinc is the one thing that will go to the apex.

Dr. Hightower learned his method from Dr. Crenshaw, but finds that it sometimes goes through the apex and causes trouble.

Dr. C. V. Rosser has not fixed upon the one best method; has occasional failures with one as with the other. Thinks some really scientific method ought to be settled upon. Wants to know how to avoid the acute trouble that sometimes follows opening up a long-time quietly dead tooth, in spite of all precautions. Would not use amalgam or cement in a root canal unless he were absolutely sure there would be no subsequent trouble, which he never is sure of.

DR. T. M. ALLEN thinks a very important point to be always taken into consideration is the age of the patient, probable degree of calcification and condition of the apex. As much care is needed not to force air through as with filling materials, instruments or medicaments. Air, forced through, expands through the heat of the tissues and causes swelling, inflammation and suppuration.

DR. H. B. WILLIAMSON fills root-canals with lead points dipped in iodoform. Even if forced through, will do no harm. Easy to fit, causes no inflammation, never fails.

THE NIGHT SESSION

was taken up in the discussion of a paper on "Mechanical Dentistry," by Dr. S. W. Foster, of Atlanta. Those who took part in the discussion were Drs. T. M. Allen, R. C. Young, J. P. Gray, George S. Vann, A. A. Pearson, W. G. Browne, William Crenshaw, C. V. Rosser, P. R. Tunstall.

DR. FOSTER said that while no branch of dental art had been so neglected and abused by the average practitioner, yet there are but few who really know how to properly execute this class of work (plate work as it is usually called). There is nothing that demands higher skill, artistic taste, and knowledge of the laws that govern, in the restoration of features, selection of teeth correct in shape, size, color and expression according to the different temperaments and their combinations. It is true that any properly trained *mechanic* can take an impression, get the bite, run the models, put together peices of rubber and blocks of teeth and give it a beautiful finish, and perhaps satisfy the patient. But all that is not prosthetic art.

For the class of mechanical work described, the full denture is the simplest piece of work, but to the prosthetic artist, it is with the full denture that it is most difficult to "make art conceal art," in restoring the natural contour to shrunken features, and in selecting teeth that shall be in harmony with the age and complexion of the patient, with the color of the hair and eyes, and typical of the temperament. This demands time, study and knowledge not recognized by the average "plate worker."

The discussion dwelt at some length upon the comparative merits of plaster, wax and modelling compound in taking impressions, each having advocates and opponents.

DR. T. M. ALLEN condemned strongly the "travelling dentist" who pulls out all the teeth in a rural community on one round and brings back the ready made "sets" in his next trip, buying his teeth by the wholesale, all of the same size and shade, regardless of individuality.

DR. R. C. YOUNG deplored the extraction of roots which should be treated and filled preserving the contour of the arch, crowning wherever possible and resorting to the plate only in the last extremity.

PROF. GRAY commends the emphasis placed upon knowledge of temperaments by the essayist, which he said, was almost a hobby with him. A knowledge of the corresponding contour, color, shape, etc., of the teeth is necessary in the selection of teeth to unite temperaments of mixed type, as are nearly all American people. He deplored the custom of turning over plate work to the student or of sending it out to some one who has not seen the patient. No matter how exquisite the workmanship may be it will not have the essential individuality. He said you have no right to do it. It is your duty to give your patients your best work in all lines, and you have no right to give it to some one else to do for you, or if you do it yourself putting it off till night when you are too tired to do it properly. It should hold a place in your estimation not excelled by any other work in your practice. You should never take an impression of the mouth without getting with it a vivid impression of the patient's whole face, with every shade of individuality, and then make the teeth to suit all the rest. Crown and bridge work is as much mechanical dentistry as is plate work. Filling cavities and restoring contour of the teeth is purely mechanical work.

DR. J. S. VANN spoke in favor of modelling compound for taking impressions in difficult cases. He said modelling compound gives a perfect impression, but it is a material that must be studied and used properly. It soon looses its plastic properties. It should be softened in warm water, and after it is placed in the impression cup, warmed again over the flame, getting the palatal surface much warmer than that next the cup. Hold it up firmly in place, not relaxing the pressure while with syringe and cold water you cool off the impression cup until, on raising the lip you feel that it is cool from above. It must feel thoroughly cool before it is ready to remove from the mouth.

Dr. A. A. Pearson. I agree with Dr. Gray that we ought to give more attention to our plate work. We ought to take hold of it in the morning when we are fresh and vigorous.

Dr. Gray. When modelling has once moved in the mouth the impression is ruined. The patient must not swallow or the soft palate pulls it down and the back part of the plate wont fit. I used it for three years and I spent the next three years in making over the plates of those of my patients who did not go some where else for a plate that would fit.

Dr. W. G. Browne gets most excellent results with modelling compound. He puts the tray in the mouth and tells the patient to bite up on it and hold it there himself.

Dr. Crenshaw spoke of the superior results from using the dry heat or superheated steam process, with the Seabury vulcanizer.

Dr. Rosser said that for plate work to be a success the case must be studied—diagnosed—as carefully as the physician diagnoses a case of serious illness. He said one cause of the unsatisfactory plates we use, is the inaccessibility of the dental depots to many men. They carry a large stock of teeth and if they have not got just what they want, use the best they have got. They may have ability and perhaps artistic teeth, but circumstances are against them.

Dr. Allen. There are competent men in the depots who will select the proper teeth if a guide for color and size is sent with proper information. The trouble is they order all their teeth from the same mold. They write, send me so many sets of teeth, with no distinction of shade or size, and they use them indiscriminately for all cases. They are mere mechanics. They may be able to get a good impression but it is doubtful if they know how to set up the teeth. They are satisfied if they can make the teeth stick in the wax 'till they get the plaster poured.

Wednesday the association spent the day on the waters of Mobile Bay and the Mexican Gulf.

The next session was called to order at 8:30 P. M. when the discussion of mechanical dentistry was continued.

DR. GEO. H. TAYLOR gave many interesting items concerning laboratory methods, refining gold scraps and making solder of a definite carat, etc. Bridge work was discussed at some length. Dr. Taylor maintained that it is impossible for any one to construct a bridge from the impression or model without seeing the patient, while Dr. Allen claims to have perfect success every time, never having had one returned as unsatisfactory, though he does not see the patient. Dr. Allen also maintained that it is poor economy for the dentist of the present day to spend his time making solder and refining gold scraps, when everything of the kind is so well done, ready for use, and kept by reliable dental depots. Dr. Taylor and Dr. Browne claim great satisfaction for the die-stamped crowns. Dr. Rosser wants an open mouth band that he can look into and see that it fits before he puts on the cusps.

DR. BROWNE stamps the cusps of a shell crown by putting modelling compound in the blank before driving down the mandrel.

DR. TAYLOR uses gold foil leaving it in the cusps, adding a little borax and fusing with blow pipe.

DR. ALLEN considers borax in soldering absolete, using Parr's flux.

DR. BROWNE stamps an aluminum blank (with the Morrison die plate), for use over a gutta percha cap molded over a tooth partially prepared for crowning, putting crystals of cocaine in the gutta percha cap to prevent the tooth from becoming hypersensitive between sittings.

DR. R. A. RUSH read a paper for discussion under "Mechanical Dentistry," describing a case, exhibiting the model, in which as the result of a pistol used with intent of suicide the superior alveolar process and teeth had been shattered and a large opening made into the antrum through the palate. The antrum is denuded of lining membrane, the bone being bare. Food and water pass into the antrum, it being necessary to introduce fluids into the throat through a funnel. It is also necessary for the poor man to hold his nose when smoking. A superior molar on each side and a central incisor are all the teeth left. A plate constructed for the case had gold clasps around the teeth and a protuberance in the plate closing the opening into the antrum. The central incisor had since been lost from pyorrhœa alveolaris and Dr. Rush desired suggestions as to the best method of constructing another plate for the case. He feared that gold clasps might eventually cause the loss of the molars, one of which is a dead tooth and penetrates into the antrum. After considerable discussion and numerous suggestions, nothing better was offered than either a good suction plate, or crowning the molars and securing the plate to the crowned teeth.

Dr. Browne would gold-line a rubber plate and anchor as above.

Dr. Allen would make the plate of black rubber, vulcanizing between tinfoil surfaces, making the clasps of soft rubber.

Subject "Mechanical Dentistry" passed.

